# Genomic Characterization of RTK-RAS Pathway Alterations in Juvenile Myelomonocytic Leukemia Through Whole-Exome Sequencing

**DOI:** 10.3390/medsci14050583

**Published:** 2026-09-18

**Authors:** Harsh Goel, Ravi Kumar Majhi, Jagdish Prasad Meena, Anita Chopra, Sameer Bakhshi, Lata Singh, Rachna Seth, Bibekananda Kar, Pranay Tanwar, Aditya Kumar Gupta

**Affiliations:** 1 Laboratory Oncology Unit, Dr. B.R.A. Institute of Rotary Cancer Hospital, All India Institute of Medical Sciences, New Delhi 110029, India; goel.harsh271@gmail.com (H.G.);; 2 Division of Pediatric Oncology, Department of Pediatrics, All India Institute of Medical Sciences, New Delhi 110029, India; 3 Department of Medical Oncology, Dr. B.R.A. Institute of Rotary Cancer Hospital, All India Institute of Medical Sciences, New Delhi 110029, India; 4 Department of Pediatrics, Masonic Cancer Center, University of Minnesota, Minneapolis, MN 55455, USA

**Keywords:** juvenile myelomonocytic leukemia, whole-exome sequencing, RTK-RAS, PTPN11, mutational signatures, therapeutic targets

## Abstract

Background/Objectives: Juvenile myelomonocytic leukemia (JMML) is a rare and very aggressive pediatric myelodysplastic/myeloproliferative neoplasm with molecular heterogeneity and constitutive activation of the RAS signaling pathway. The aim of this study was to identify the mutational landscape, driver genes, mutational signatures, functional pathways, and therapeutic targets of mutations that affect receptor tyrosine kinases (RTKs) and RAS pathways in JMML. Methods: We collected tumor and matched buccal swab samples from 35 patients with JMML and performed whole-exome sequencing. Variants were called using GATK-Mutect2 and annotated with ANNOVAR. Mutational profiling, co-occurrence analysis, protein domain mapping, and driver gene identification were performed using maftools and OncodriveCLUST. DGIdb was used to explore drug–gene interactions, MutationalPatterns was used for the characterization of mutational signatures, and the clusterProfiler package was used for the functional enrichment analyses. Results: Alteration of the RTK-RAS pathway was found in 77.1% of patients. *PTPN11* (23%), *NRAS* (20%), and *KRAS* (14%) were the most frequently altered genes, followed by *PTEN* (11%) and *FLT3*, *ROS1*, *FGFR4*, *ERBB2*, and *EGFR* (9% each). Domain mapping of protein sequences identified the grouping of mutations within the conserved functional domains of the proteins. The alterations in receptor tyrosine kinase genes demonstrated a significant degree of co-occurrence, while the alterations in canonical JMML genes showed mutual exclusivity. *PTPN11*, *NRAS*, *ROS1,* and *FGFR4* were top driver genes based on driver gene analysis. Functional enrichment analysis revealed significant enrichment of receptor tyrosine kinase signaling, Ras/MAPK and PI3K-AKT pathways. The mutational signatures were mostly SBS5-like, with enrichment of C>T transitions, indicative of endogenous mutational processes. Drug–gene interaction analysis identified *EGFR*, *ALK*, *ROS1*, *ERBB2*, *FLT3*, *FGFR4*, and *PDGFRA* as highly interconnected and potentially actionable therapeutic targets. Conclusions: These findings expand the molecular landscape of JMML and underscore the importance of aberrant RTK-RAS signaling in disease pathogenesis. The identification of recurrent and potentially druggable RTK alterations provides a rationale for future precision medicine approaches in JMML.

## 1. Introduction

Juvenile myelomonocytic leukemia (JMML) is a rare and highly aggressive clonal hematopoietic disorder of early childhood caused by transformation of haematopoietic stem/progenitor cells (HSPCs) and occurs in about 1.2 cases per million children per year, with a median age at diagnosis of 2 years (range 0–14 years) [1,2]. The incidence of JMML is about 1% of all pediatric leukemias and 20% to 40% of pediatric myelodysplastic syndromes (MDS), and is more common in males (the male-to-female ratio is approximately 2:1) [3]. The fifth edition of the World Health Organization (WHO) Classification of Tumours reclassified JMML as a myeloproliferative neoplasm, while the International Consensus Classification (ICC) adopted JMML under pediatric and/or germline mutation-associated disorders [4,5]. In most cases, JMML is associated with clinical features of bone marrow and organ infiltration by malignant mature and immature myeloid cells [6]. JMML is associated with hyperproliferation of both monocytic and granulocytic lineages, abnormal responsiveness of myeloid progenitors to GM-CSF (granulocyte-macrophage colony-stimulating factor), splenomegaly and clinically variable outcomes [7,8,9]. Chromosomal studies of leukemic cells show monosomy 7 in 25% of JMML patients, with other abnormalities in 10%, but the majority of patients (65%) have a normal karyotype [10]. Although allogeneic hematopoietic stem cell transplantation (HSCT) remains the only curative treatment option, relapse occurs in approximately one-third of patients, emphasizing the need for improved molecular characterization and targeted therapeutic strategies [11,12]. The molecular pathogenesis of JMML is primarily driven by hyperactivation of the RAS signaling pathway. Constitutive activation of RAS signaling in hematopoietic stem and progenitor cells results in persistent downstream signaling through the RAF-MEK-ERK and PI3K-AKT pathways, promoting abnormal cellular proliferation, enhanced survival, and impaired differentiation. This leads to hypersensitivity of myeloid progenitors to granulocyte-macrophage colony-stimulating factors (GM-CSFs), uncontrolled expansion of the myelomonocytic compartment, and clonal hematopoiesis, thereby contributing to neoplastic transformation. Additional cooperating genetic and epigenetic alterations may further promote clonal evolution, disease progression, and clinical heterogeneity. Nearly 90% of cases harbor canonical mutations in PTPN11, NRAS, KRAS, NF1, CBL and these mutations lead to constitutive activation of the RAS/MAPK signaling cascade. The 5 canonical RAS pathway alterations delineate 5 genetically and clinically distinct JMML subtypes. Two subtypes of JMML are associated with germline RAS disease (in children with neurofibromatosis type 1) and biallelic inactivation of tumor suppressor genes (in children with CBL syndrome) in hematopoietic cells, and three subtypes are associated with heterozygous somatic gain-of-function (GOF) mutations in non-syndromic children; PTPN11, NRAS, and KRAS-mutated JMML [13]. These genetic abnormalities result in aberrant proliferation and differentiation of hematopoietic progenitors. Mutations in several RAS pathway genes have also been linked to developmental disorders, termed RASopathies, which increase susceptibility to JMML, further supporting the importance of dysregulated RAS signaling in disease initiation and progression [14,15]. These changes include mutations in PTPN11, the most frequent molecular event, which is linked to a more aggressive disease phenotype and poorer clinical outcome [16]. Similarly, the mutations in NRAS or KRAS result in activation of the GTPase and downstream signaling mechanisms in the RAF-MEK-ERK and PI3K-AKT pathways, respectively [17,18]. Loss of NF1 and CBL also leads to constitutive activation of the RAS pathway and increased responsiveness to growth factors [19,20,21]. Despite these advances, there is still a lot of genetic diversity among patients, and other cooperating mutations in receptor tyrosine kinases (RTKs) and downstream signaling molecules may play a role in disease evolution and therapeutic resistance [22]. Recent advances in next-generation sequencing technologies have substantially improved the understanding of the genomic landscape of JMML [23]. Beyond improving the detection of disease-associated genetic alterations, NGS-based molecular characterization has the potential to refine diagnostic classification, identify genetically defined subgroups, and support prognostic stratification. However, the clinical utility of molecular subclassification depends on its ability to provide additional diagnostic or prognostic information beyond established clinicopathological parameters. Whole-exome sequencing (WES) studies have identified recurrent secondary mutations involving epigenetic regulators, spliceosome genes, transcription factors, and signaling molecules that influence disease progression and prognosis [24]. Furthermore, treatment resistance or relapse is associated with clonal evolution and acquisition of subclonal mutations, highlighting the need for in-depth genomic characterization [25]. Receptor tyrosine kinases are important mediators of extracellular signaling and activate several pathways such as RAS/MAPK, PI3K/AKT and JAK/STAT signaling [26]. The dysregulations of RTKs are widely recognized in solid tumors and hematological malignancies, with mutations in genes like FLT3, EGFR, ERBB2, ALK, ROS1, FGFR4 and PDGFRA involved in oncogenesis and making them clinically relevant targets for therapeutic intervention [27,28]. Although canonical RAS pathway mutations are well established in JMML, the broader spectrum of RTK-associated alterations and their contribution to disease biology remain incompletely understood. In the current study, whole-genome exome sequencing was carried out on paired samples of bone marrow and matched buccal swabs from JMML patients for comprehensive characterization of the alterations in the RTK-RAS signaling network.

## 2. Materials and Methods

### 2.1. Patient Sample Collection and Whole-Exome Sequencing

Bone marrow (BM) samples were collected from 35 patients with juvenile myelomonocytic leukemia (JMML) with written informed consent from their parents/legal guardians, along with matched buccal swab samples. All patients included in this study fulfilled the applicable diagnostic criteria for JMML according to both the WHO 5th edition and ICC 2022 classification systems. The study was done in adherence to the guidelines of the Declaration of Helsinki and was approved by the Institutional Ethics Committee, All India Institute of Medical Sciences (AIIMS), New Delhi, India (Approval No: IEC-351/08.5.2020/RP-41/2020; Approval Date: 21 May 2020). The BM samples were processed by Ficoll-Paque density gradient centrifugation to isolate mononuclear cells. For all 35 patients, matched buccal swabs were collected and used as germline controls during somatic variant calling. For the purposes of this study, variants identified in the BM sample and classified as somatic by the matched tumor-normal variant-calling approach using Mutect2 were included in the somatic mutation analysis, whereas variants detected in the matched buccal sample were not included as somatic variants. Although matched buccal swabs were used as non-tumor controls, constitutional mosaicism and potential contamination of buccal DNA by hematopoietic cells cannot be completely excluded. Genomic DNA was isolated from each sample using the DNeasy Blood and Tissue Kit (Qiagen, Hilden, Germany) according to the manufacturer’s instructions. DNA concentration was measured with a Qubit Fluorometer, and DNA integrity and purity were evaluated by the Agilent TapeStation 4200 system (both from Agilent Technologies, Santa Clara, CA, USA). Samples with good DNA quality (A260/280 between 1.8 and 2.0) were chosen for library preparation. Libraries for whole-exome sequencing (WES) were constructed following manufacturer’s instructions with the Agilent SureSelect Human All Exon V7 Kit (Agilent Technologies, Santa Clara, CA, USA). Sequencing was carried out using a paired-end sequencing chemistry (2 × 150 bp) on the Illumina NovaSeq 6000 platform (Illumina, San Diego, CA, USA). Bone marrow samples were sequenced with a target sequencing depth of approximately 250×, whereas matched buccal swab samples used as germline controls were sequenced with a target depth of approximately 80–100×.

### 2.2. Somatic Variant Calling and Annotation

Sequencing reads were obtained from raw WES data, and quality checks were performed using FastQC (v0.12.1). Trimmomatic (v0.39) was used to remove adapter sequences and low-quality bases. Burrows-Wheeler Aligner MEM (BWA-MEM v0.7.17) was used to align high-quality reads to the human reference genome (hg19). All post-alignment processing was carried out according to best-practice guidelines as recommended by the Genome Analysis Toolkit (GATK v4.4). Matched buccal swab DNA samples were used as germline controls to differentiate variants from inherited polymorphisms. Somatic single nucleotide variants (SNVs) and small insertions/deletions (indels) were detected with Mutect2 and filtered with FilterMutectCalls. Variants detected in matched buccal swab samples were excluded from further analyses. Variants were annotated using ANNOVAR (version 2020-06-08) against the hg19 reference genome. Population frequency and clinical databases included dbSNP build 151, ClinVar (release dated 11 June 2024), COSMIC version 70, gnomAD exomes version 2.1.1, ESP6500SI-V2, and the 1000 Genomes Project Phase 3 database (August 2015 release). Functional and splice-site annotations were obtained using dbNSFP version 4.7a and dbscSNV version 1.1, respectively. Variants with a population allele frequency ≥1% in gnomAD exomes version 2.1.1, ESP6500SI-V2, or the 1000 Genomes Project Phase 3 database were considered common population variants and excluded from downstream somatic variant analyses. For downstream somatic variant interpretation, variants with a variant allele frequency (VAF) below 10% were excluded, as variants below this threshold were outside the scope of the assay. The maftools package in R (v4.3.1) was used to generate and analyze Mutation Annotation Format (MAF) files.

### 2.3. RTK-RAS Mutational Landscape Analysis

Following comprehensive genome-wide somatic variant calling and annotation of the whole-exome sequencing data, the annotated variants were further evaluated for downstream pathway-specific analyses. For the present study, we focused on genes involved in receptor tyrosine kinase (RTK) signaling, RAS signaling, downstream pathway regulation, and related adaptor proteins. These genes were compiled based on published studies of JMML and the Kyoto Encyclopedia of Genes and Genomes (KEGG) and Reactome pathway databases. The annotated MAF files were subsequently used to identify high-confidence variants in RTK-RAS pathway-associated genes for detailed downstream analyses. maftools was used to summarize mutation frequencies, variant classifications, variant types, and mutation burden. MAF summary analyses were performed to characterize the overall mutational profile of the cohort. Oncoplots were created to illustrate the distribution of alterations in the RTK-RAS pathway for each of the 35 JMML samples. The frequency of mutations was determined based on the percentage of patients with at least one somatic mutation in a particular gene. Further analyses of nucleotide substitution patterns were conducted to assess mutational spectra and mutational heterogeneity within the cohort, such as transition/transversion (Ti/Tv) analyses. To characterize mutational spectra, single-nucleotide variants were classified into six base-substitution classes: C>T, C>G, C>A, T>C, T>G, and T>A. C>T and T>C substitutions were classified as transitions, whereas C>A, C>G, T>A, and T>G substitutions were classified as transversions. The frequencies of individual substitution classes and the transition/transversion ratio were assessed at both the cohort and sample levels. The Ti/Tv ratio was calculated as the total number of transition events divided by the total number of transversion events among high-confidence RTK-RAS pathway SNVs.

### 2.4. Protein Domain Mapping and Mutation Visualization

The position of recurrent mutations within the protein domains of RTK-RAS pathway genes was analyzed using the lollipopPlot function in maftools. Protein domain annotations were generated from the RefSeq transcript and amino acid coordinate information. Mutated genes were mapped onto the protein structures, including PTPN11, NRAS, KRAS, FLT3, FGFR4, ROS1, ERBB2, EGFR, ALK and PDGFRA, to find mutation hotspots and functional domains. The mutations in conserved domains were evaluated to determine possible deleterious changes in protein function and signaling activity which could contribute to pathogenicity.

### 2.5. Co-Occurrence, Mutual Exclusivity, and Variant Allele Frequency Analysis

The Somatic Interactions function provided by maftools was used to explore patterns of mutational interaction among repeatedly altered genes in the RTK-RAS pathway. Fisher’s exact test was used for pairwise associations. A positive association was interpreted as co-occurring mutations, and negative association as mutually exclusive events. A *p*-value < 0.05 was considered statistically significant, while associations with *p*-values ≥ 0.05 were considered not statistically significant. Given the relatively small cohort size, sparse mutation events for several genes, and the number of pairwise comparisons performed, the interaction analysis was considered exploratory, and statistically significant associations were interpreted cautiously. Interaction strengths were displayed as the −log10 (*p*-values). MAF files were used to extract variant allele frequencies (VAFs) and to assess the clonal representation of recurrent RTK-RAS mutations. VAFs were plotted as boxplots across the genes that are recurrently altered, allowing for comparison of the mutation burden and possible architecture of the growing clone within the altered JMML samples. Copy-number alterations, loss of heterozygosity, and tumor purity were not incorporated into the VAF analysis. Therefore, VAF was interpreted as a descriptive measure of allelic representation and was not used as a definitive estimate of cancer cell fraction or to establish clonal or subclonal architecture.

### 2.6. Driver Gene Identification and Clustering Analysis

OncodriveCLUST was used to identify candidate driver genes by identifying genes with significant clustering of mutations in specific protein regions. Permutation-based statistical testing was used to assess mutation clustering significance, while false discovery rate (FDR)-adjusted q-values were computed. Genes demonstrating significant positional clustering were considered potential driver genes. Bar plots and clustering analysis were used to summarize mutation counts and frequencies, to show the proportion of samples with mutations, and to infer clustering significance to identify the major RTK-RAS pathway drivers in the JMML cohort. Given the relatively small cohort size and the low mutation frequency of several genes, the results of the OncodriveCLUST analysis were interpreted as exploratory computational evidence for putative driver candidates and not as definitive evidence of driver function or oncogenic activity.

### 2.7. Drug–Gene Interaction and Therapeutic Target Analysis

The Drug–Gene Interaction Database (DGIdb v4.3.0) was used to find potentially actionable therapeutic targets. Recurrently mutated genes in the RTK-RAS pathway were analyzed for known and predicted drug interactions to uncover clinically relevant therapeutic opportunities. Drug–gene interaction networks were created and displayed using igraph (v2.0.3) and Cytoscape (v3.10.2). The genes were also categorized based on the functional classes in the DGIdb, such as clinically actionable genes, kinases, phosphatases, receptor-associated proteins, tumor suppressors, signaling regulators, and druggable genome components. Bar plots and network-based visualizations were created for the number of therapeutic interactions related to each gene and the distribution of druggable functional categories. Only curated and pharmacologically relevant interactions were retained for downstream interpretation.

### 2.8. Mutational Signature Analysis

Mutational signature analysis was carried out using MutationalPatterns (v3.18) to define mutational processes occurring in JMML. Somatic SNVs from MAF files were grouped into the 96 trinucleotide substitution contexts. Non-negative matrix factorization (NMF) was used to extract de novo mutational signatures. The extracted signatures were subsequently compared with the COSMIC v3.3 reference mutational signature database using cosine similarity analysis. Given the limited availability of JMML-specific mutational signature data in COSMIC, this comparison was performed to assess the similarity of mutational patterns observed in our cohort to established COSMIC mutational signatures and was not intended as a JMML-specific reference comparison. The signature contributions across samples were displayed in stacked bar plots, and the cosine similarity heatmap was created to determine the most similar COSMIC single-base substitution (SBS) signatures and their associated biological causes.

### 2.9. Functional Enrichment and Pathway Analysis

The top 20 most frequently mutated genes identified in our cohort that were associated with the RTK-RAS signaling pathway were selected for functional enrichment and pathway analysis. Gene Ontology (GO) enrichment analysis was performed using the clusterProfiler package in R software across three functional categories: Biological Process (BP), Molecular Function (MF) and Cellular Component (CC). Pathway enrichment analyses were also performed using the Reactome and Kyoto Encyclopedia of Genes and Genomes (KEGG) databases to characterize the functional and biological pathway associations represented by these 20 genes. Hypergeometric testing with Benjamini–Hochberg multiple-testing correction was used to assess statistical significance. Pathways that had an FDR adjusted *p*-value < 0.05 were considered significant. Bubble plots were used to visualize enrichment results showing enrichment significance, gene count, and pathway-level association. Because the input genes were preselected based on their mutation frequency and association with RTK-RAS signaling, the enrichment results were interpreted as a functional characterization of the selected gene set and its associated pathways and not as independent evidence of pathway activation in JMML samples.

### 2.10. Statistical Analysis

All statistical data analyses and visualizations were carried out using R software version 4.3.1 and Python version 3.14.5. Median and range values were used for continuous variables, and frequencies and percentages were used for categorical variables. Pairwise mutation associations were tested using Fisher’s exact test for co-occurrence analyses. Multiple testing correction was performed using the Benjamini–Hochberg procedure, and adjusted *p*-values (FDR) < 0.05 were considered statistically significant throughout the study.

## 3. Results

### 3.1. Somatic Mutation Landscape of RTK-RAS Pathway Genes in JMML

**Figure 1:**
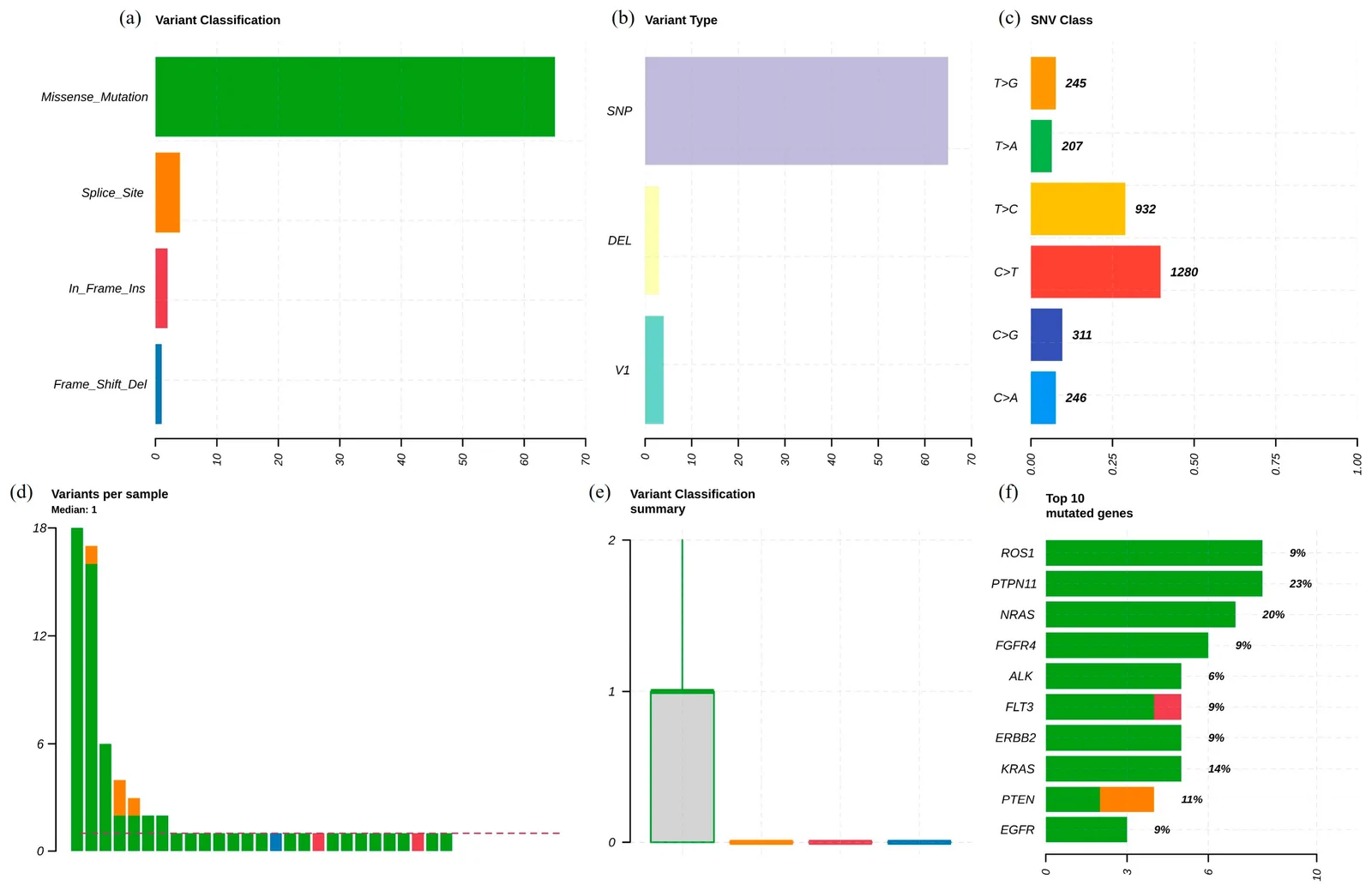
Mutational landscape of RTK-RAS pathway genes in JMML patients. (**a**) Classification of variants in the RTK-RAS pathway. (**b**) Distribution of types of variants. (**c**) Single-nucleotide variant (SNV) substitution spectrum. (**d**) Distribution of RTK-RAS pathway variants per sample. (**e**) Frequencies of variant classification. (**f**) The most frequently mutated RTK-RAS pathway genes and their mutation frequencies in the 35 samples of JMML.

Following comprehensive analysis and annotation of the whole-exome sequencing data, the present study focused on the characterization of alterations affecting the RTK-RAS signaling network, given its established central role in JMML pathogenesis. Accordingly, the detailed results presented below focus on variants identified in RTK-RAS pathway-associated genes. The most common type of mutation in the RTK-RAS pathway were missense mutations (Figure 1a). To determine the nature of genomic changes, a variety of variants was analyzed, and it was found that the vast majority of genomic changes were single nucleotide polymorphisms (SNPs), with fewer insertions and deletions (Figure 1b). The examination of the spectrum of single nucleotide substitutions indicated that C>T transitions (1280) were more prevalent, followed by T>C substitutions (932), and C>G, C>A, T>G and T>A substitutions were less abundant (Figure 1c). The median number of mutations in the RTK-RAS pathway was one per sample, suggesting that the majority of patients had a small number of recurrent RTK-RAS lesions (Figure 1d). PTPN11 was the gene most frequently mutated in patients with recurrently altered genes (8/35; 23%), followed by NRAS (7/35; 20.0%) and KRAS (5/35; 14%) (Figure 1f). Additional recurrent alterations were identified in PTEN (4/35, 11%), ROS1, FGFR4, FLT3, ERBB2, and EGFR (3/35 each, 9%), whereas ALK mutations were observed in two patients (5.7%). These findings validate the predominance of the canonical JMML-associated RAS-pathway lesions, including PTPN11, NRAS, and KRAS, and also highlight the recurrent members of the receptor tyrosine kinases and associated signaling regulators: ROS1, FGFR4, EGFR, ERBB2, FLT3, and ALK. Together, these results underscore the importance of RTK-RAS pathway dysregulation in JMML pathogenesis and detail the mutational landscape of the pathway across the cohort.

### 3.2. RTK-RAS Pathway Alterations Across the JMML Cohort

**Figure 2:**
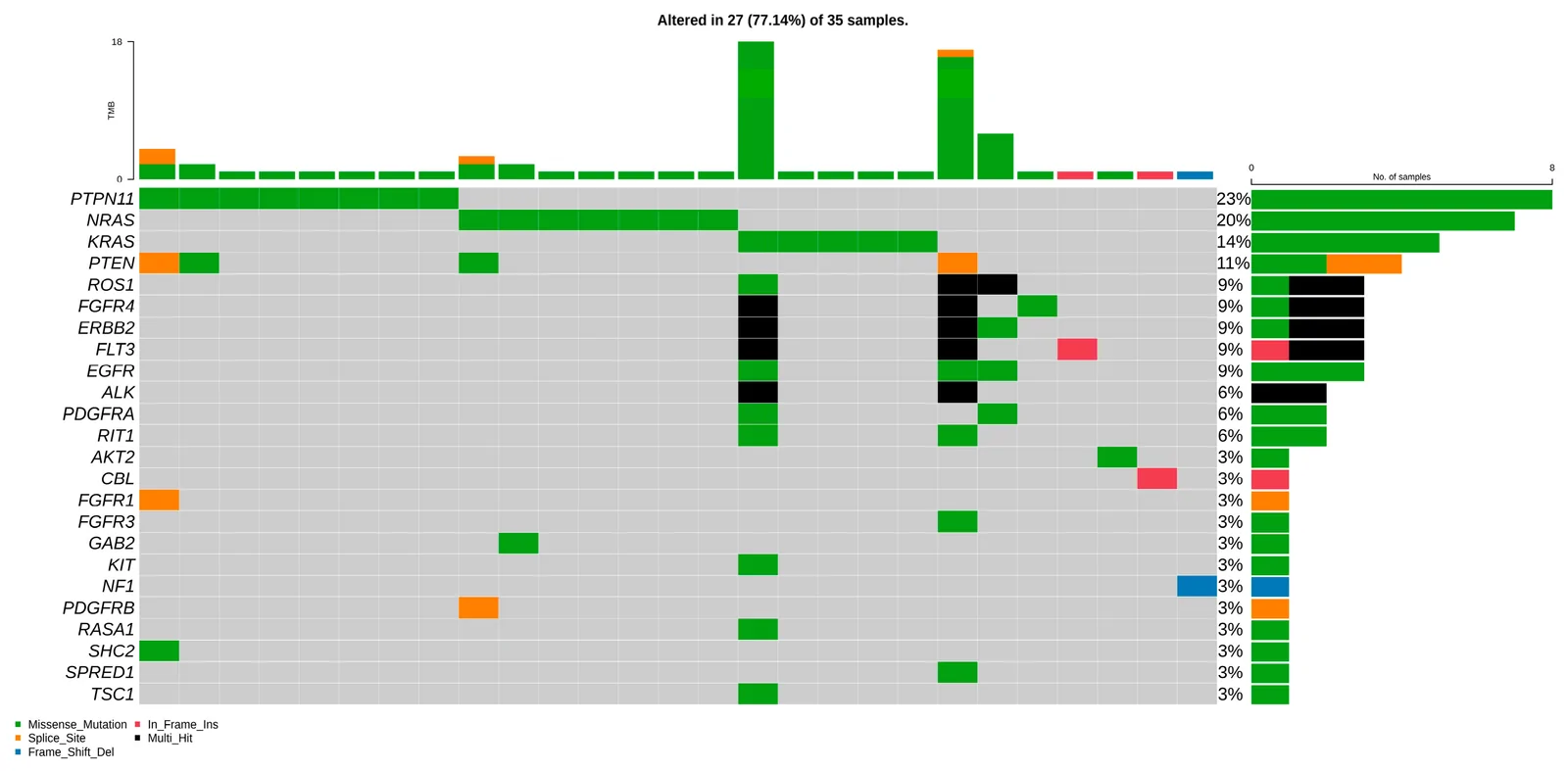
Alterations of the RTK-RAS Pathway in JMML Cohort. Oncoplot shows the distribution of somatic alterations in RTK-RAS pathway genes in 35 JMML patients. Columns represent individual patients and rows represent recurrently altered RTK-RAS pathway genes. The upper bar plot shows the number of mutations in each sample (tumor mutational burden, TMB); the right-side bar plot shows the frequency of each mutation across genes. Mutation classes are color-coded and include missense mutations, splice-site variants, frame-shift deletions, in-frame insertions, and multi-hit events.

The distribution of RTK-RAS pathway alterations was explored per patient by creating an oncoplot based on the whole-exome sequencing data set of JMML patients (Figure 2). In total, 27/35 patients (77.1%) had at least one somatic alteration in an RTK-RAS pathway-associated gene, indicating the widespread central role of this pathway in JMML pathogenesis. PTPN11 was most commonly mutated (eight of 35 patients, 23%), followed by NRAS (seven of 35 patients, 20%) and KRAS (five of 35 patients, 14%), as is typical of the mutational landscape. Other recurrent mutations included PTEN (11%), FGFR4 (9%), ERBB2 (9%), FLT3 (9%), and EGFR (9%). Mutations in ALK, PDGFRA and RIT1 were found in 6% of patients, further underlining the genetic variety of the dysregulated RTK-RAS pathway in JMML. Missense variants were the most common mutation type, while few splice-site variants, frame-shift deletions, and in-frame insertions were found. Multiple receptor tyrosine kinases and downstream effectors were altered in several patients, indicating that multiple alterations may cooperate in the activation of the RTK-RAS pathway. Importantly, the majority of the pathway changes in the cohort were combinations of canonical driver genes of JMML (PTPN11, NRAS, KRAS, NF1, CBL, RIT1). These results define RTK-RAS signaling as a predominant molecular abnormality in JMML.

### 3.3. Transition–Transversion Pattern Analysis of RTK-RAS Pathway Mutations in JMML

**Figure 3:**
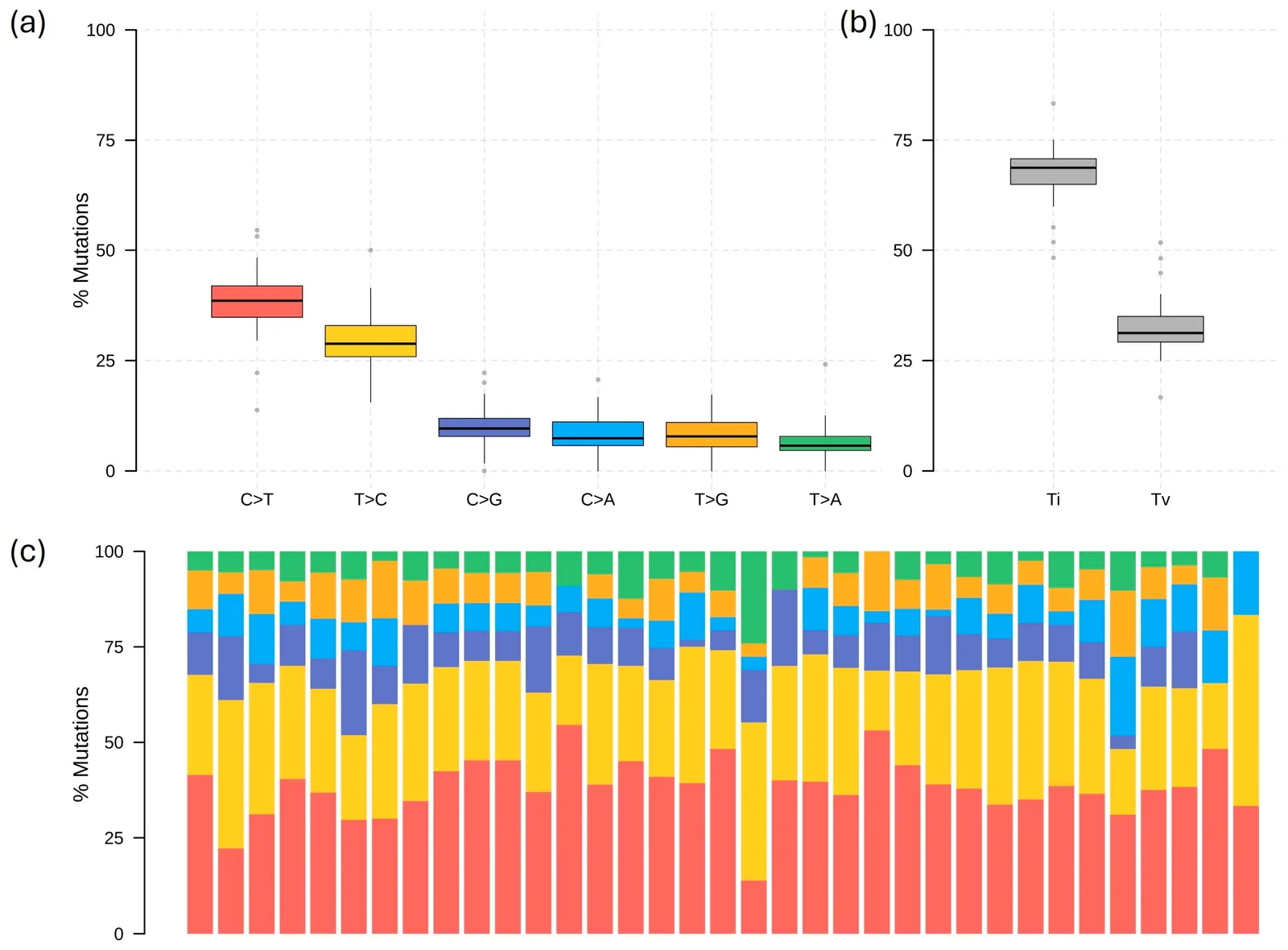
Patterns of transition and transversion of the RTK-RAS pathway mutations in JMML. (**a**) Frequency of six classes of single nucleotide substitutions in the JMML cohort. Boxplots show the percentage of each substitution type in the mutational profile of individual patients. (**b**) Overall transition (Ti) and transversion (Tv) frequencies showing a higher proportion of transition events in RTK-RAS pathway mutations. (**c**) Sample-wise distribution of nucleotide substitution classes represented in a stacked bar plot that displays the relative proportion of each substitution type in each of the 35 JMML cases.

The mutational spectrum of the changes in the RTK-RAS pathway was further characterized by analyzing the transition and transversion (Ti/Tv) patterns in all 35 patients (Figure 3). Single nucleotide substitution class analysis showed that the most common substitution type in the cohort was the C>T transition (Figure 3a), while the frequencies of C>G, C>A, T>G, and T>A substitutions were significantly lower. There was moderate inter-patient variance, but the distribution of substitution classes was consistent across samples. Overall transition and transversion frequencies showed a strong excess of transitions compared with transversions (Figure 3b). This predominance of transitions was consistently observed across the cohort, indicating a characteristic mutational pattern within RTK-RAS pathway genes. Sample-wise analysis of the nucleotide substitution spectrum also revealed that patient mutations were mostly concentrated in the two classes of C>T and T>C substitutions, while the other substitution classes contributed relatively small proportions of the mutational profile (Figure 3c). The overall substitution spectrum was well conserved across the cohort, although some variation was observed in the relative contribution of the individual substitution classes among samples. In summary, these results indicate that mutations affecting the RTK-RAS signaling pathway in JMML are highly enriched for transition-type mutations, especially C>T substitutions, indicating that common endogenous mutational processes are responsible for the acquisition of somatic alterations in this signaling pathway. These findings provide a descriptive characterization of the nucleotide substitution spectrum in the cohort and may contribute to understanding the mutational characteristics of RTK-RAS pathway alterations; however, the Ti/Tv pattern alone does not establish a specific underlying mutational mechanism or provide direct clinical utility.

### 3.4. Protein Domain Mapping and Mutation Distribution of Recurrently Altered RTK-RAS Pathway Genes in JMML

**Figure 4:**
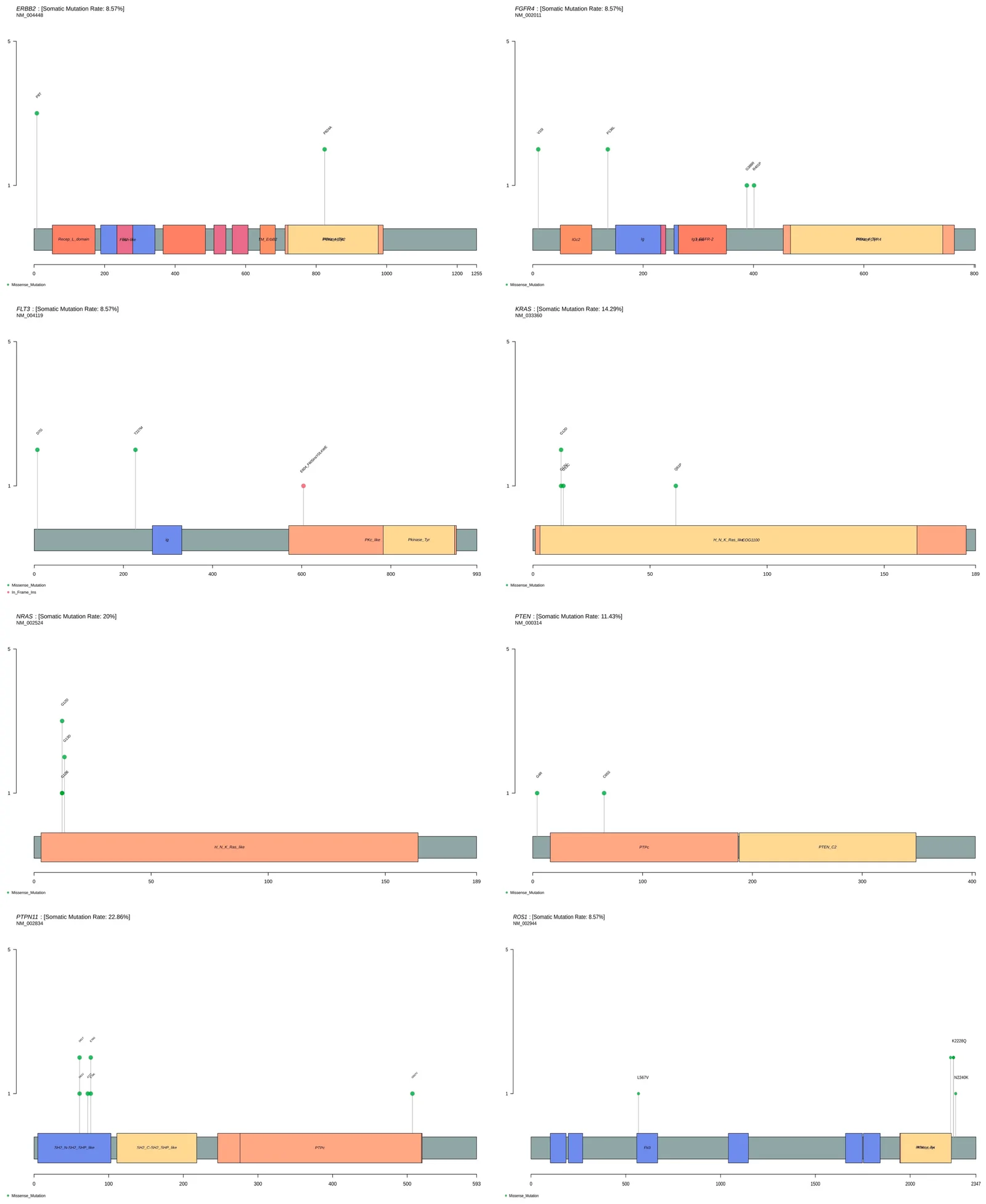
Protein domain mapping and distribution of recurrent RTK-RAS pathway mutations in JMML. Lollipop plot illustrates the distribution of somatic mutations throughout protein domains of genes frequently mutated in the JMML cohort in RTK-RAS pathway genes. Protein domains are represented by colored regions, and individual lollipops indicate amino acid substitutions detected in the cohort.

The distribution of somatic mutations was also analyzed at the protein domain level using lollipop plots (Figure 4) to further characterize the functional consequences of recurrent RTK-RAS pathway alterations. The most frequent mutations were identified in PTPN11 (22.9%), NRAS (20.0%) and KRAS (14.3%), PTEN (11.4%), FLT3 (8.6%), ROS1 (8.6%), FGFR4 (8.6%), and ERBB2 (8.6%). PTPN11 mutations were found in the N-SH2 domain, including mutations such as E76K, E76G, and D61V. NRAS contains mutations in the conserved RAS domain such as G12D and G13D, both of which are known activating mutations in the RAS pathway. Similarly, KRAS mutations were also restricted to this region and encompassed the canonical activating mutations including G12D. PTEN mutations were found throughout the functional regions of the protein that participate in phosphatase activity and membrane association, such as G4R and C65S, indicating potential disruption of tumor suppressor function among the signaling regulators. FLT3 mutations were found, including mutations like T227M and D7G that may affect the activation of the receptor and the activation of the downstream reaction. The mutations in ROS1 were found throughout multiple functional domains such as the kinase-associated domains and included L576V, K2228Q, and N2240K. Mutations in FGFR4 included substitutions (V160I, P136L, R401P), indicating different mechanisms of receptor dysregulation. Similarly, ERBB2 mutations were found throughout the functional domains, including P8T and P824A, which could suggest involvement in the regulation of aberrant receptor signaling. Altogether, protein domain mapping revealed that recurrent mutations in the RTK-RAS pathway tend to target conserved functional domains of the proteins, which are associated with the activity of a phosphatase, a GTPase, receptor signaling and kinase activation. Established activating mutations in PTPN11, NRAS and KRAS, as well as frequent alterations in receptor tyrosine kinases and regulators of the signaling pathways, further corroborate the central role of RTK-RAS pathway dysregulation in JMML pathogenesis. The localization of variants within known functional protein domains provides information regarding their structural position; however, domain localization alone does not establish the functional, oncogenic, or pathogenic significance of individual variants. Therefore, particularly for uncommon alterations identified in a limited number of patients, their potential biological relevance should be interpreted cautiously and requires further functional and clinical validation.

### 3.5. Somatic Interaction Analysis of RTK-RAS Pathway Alterations in JMML

**Figure 5:**
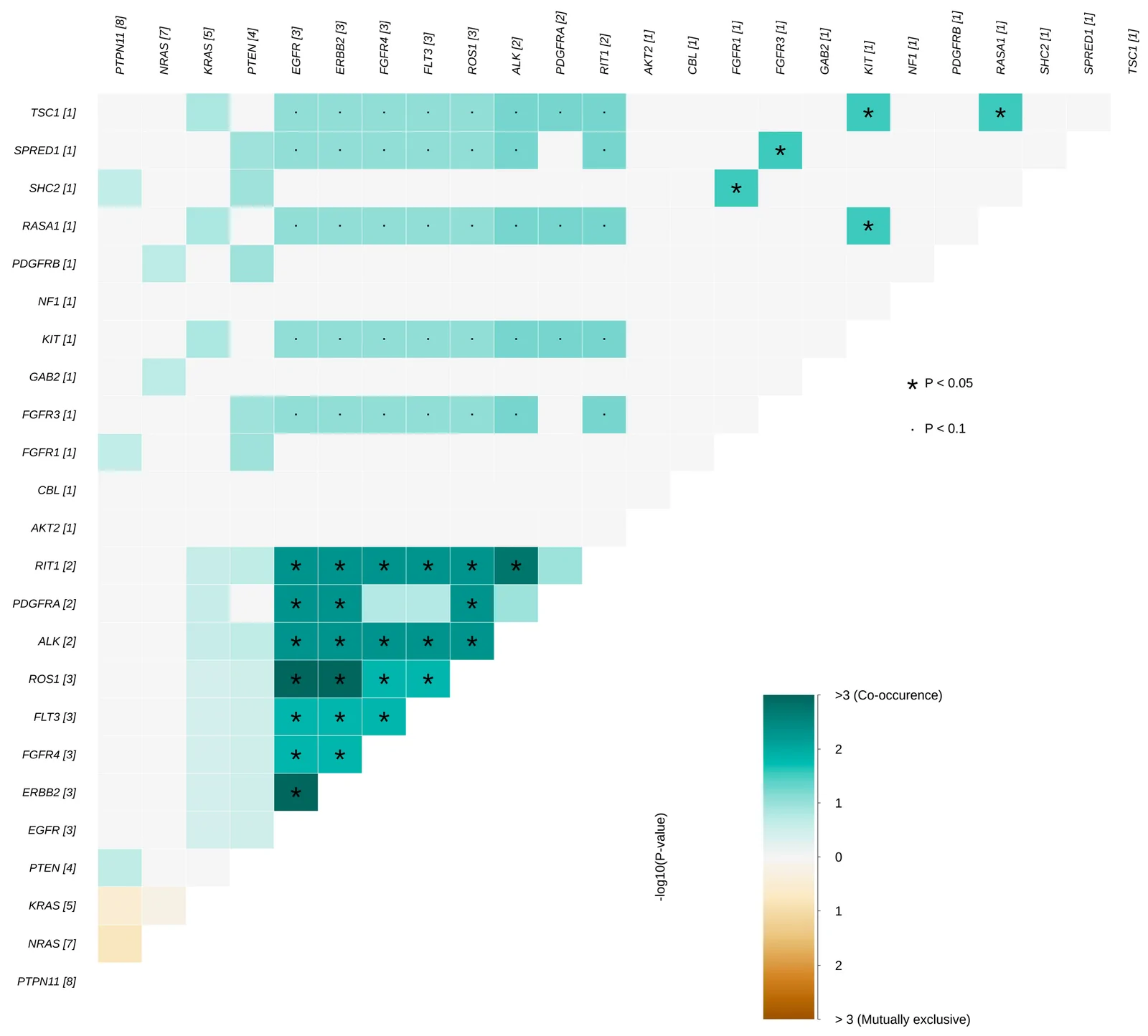
Somatic interaction landscape of RTK-RAS pathway genes in JMML. Pairwise interaction of somatically altered RTK-RAS pathway genes in the JMML cohort. The matrix shows co-occurrence and mutual exclusivity among pairs of genes. Positive associations (co-occurrence) are represented by green shading, and negative associations (mutual exclusivity) are represented by brown shading. The intensity of the colors reflects the level of significance of the interaction (as determined by the −log10(*p*-value)). Statistically significant interactions (*p* < 0.05) are marked with asterisks (*) and trends toward significance (*p* < 0.1) are marked with dots (•).

To investigate patterns of cooperation and mutual exclusivity among altered RTK-RAS pathway genes, pairwise somatic interaction analysis was performed across the 35 JMML samples (Figure 5). The interaction matrix identified a matrix of co-occurring mutations among components of the receptor tyrosine kinase, downstream signaling and regulatory pathway in JMML, indicating that there may be multiple genetic events that act cooperatively to promote the dysregulation of the RTK-RAS pathway. However, because several genes were altered in only two or three patients, the results of pairwise statistical testing should be interpreted cautiously, as associations based on sparse mutation events may be unstable. Notably, the genes ROS1, FGFR4, FLT3, ERBB2, ALK, PDGFRA, and RIT1 exhibited high positive co-occurrence values with several gene pairs showing statistical significance (*p* < 0.05). Of these, ROS1-ERBB2, ROS1-ALK, FGFR4-ALK, FLT3-ROS1, PDGFRA-ERBB2 and RIT1-ROS1 had some of the strongest co-occurrence signals within the cohort. Following Benjamini–Hochberg correction for multiple pairwise comparisons, four gene pairs remained statistically significant: ERBB2-EGFR, ROS1-EGFR, ROS1-ERBB2, and RIT1-ALK (FDR-adjusted *p* < 0.05; Supplementary Table S2). Given the relatively small cohort size, low mutation frequencies of several genes, and the multiple pairwise comparisons performed, these associations are considered exploratory and should not be interpreted as evidence of biological cooperation, functional interaction, or cooperative activation of RTK-RAS signaling. Other gene pairs showed positive co-occurrence patterns but did not meet the predefined threshold for statistical significance and are therefore described as not statistically significant. Associations with *p*-values between 0.05 and 0.10 were not interpreted as representing a trend toward significance. Patterns of mutual exclusivity were also observed among canonical JMML-associated genes. Overall, the somatic interaction analysis provides an exploratory description of patterns of joint occurrence and mutual exclusivity among RTK-RAS pathway-associated alterations. Validation in larger independent JMML cohorts will be required to determine the robustness and potential biological relevance of these observed patterns.

### 3.6. Variant Allele Frequency Distribution of Recurrently Mutated RTK-RAS Pathway Genes in JMML

**Figure 6:**
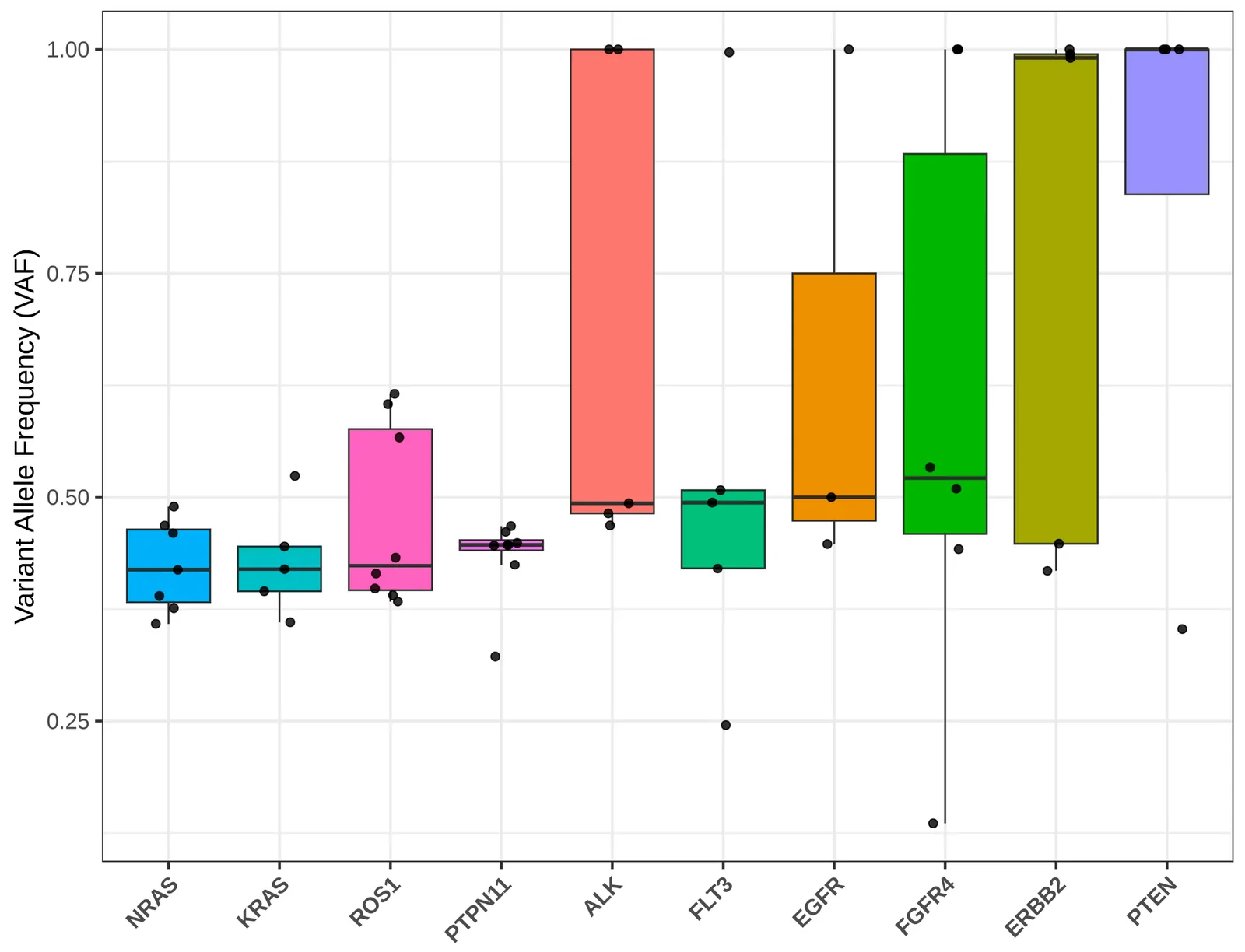
Variant Allele Frequency Distribution of Recurrent RTK-RAS Pathway Mutations in JMML. Boxplots represent the distribution of variant allele frequencies (VAFs) of recurrently mutated genes involved in the RTK-RAS pathway in the JMML cohort. Individual mutations are indicated as points and boxplots show the median, interquartile range, and distribution of all VAF values for each gene.

The distribution of variant allele frequencies (VAFs) of the most commonly mutated genes in the JMML cohort was compared to assessing the clonal representation of RTK-RAS pathway mutations (Figure 6). There was a large inter-gene and inter-patient difference in VAFs, reflecting the variation in clonal structure and mutational burden. The VAF distributions of canonical JMML driver genes PTPN11, NRAS and KRAS were relatively uniform with a median of around 40–45%. Most mutations in these genes had moderate VAFs, indicating they are dominant clonal events that are present in a significant fraction of leukemic cells. NRAS and KRAS showed relatively small VAF variations, whereas PTPN11 showed slightly larger variations, which indicated different clonal expansions in affected patients. The allelic frequency of FLT3 mutations was widely distributed from low to high, with a median VAF of about 50%, indicating significant clonally heterogeneous presentation among patients. EGFR mutations also showed a range of VAFs, ranging from around 45 to 100%, reflecting both subclonal and highly dominant mutations. Interestingly, several receptor tyrosine kinase genes had significantly high allele frequencies. ERBB2 mutations were predominantly high, with the majority of alterations occurring at frequencies over 80%, indicating that the mutations had strong clonal dominance and/or that the wild-type allele may have been lost or that copy number events may have occurred at this locus. The wide variations in VAF for ALK, FGFR4, ERBB2, and PTEN indicate significant clonally heterogeneous and time-dependent acquisition of these mutations in JMML. In summary, VAF analysis showed a range of clonal abundances for recurrent RTK-RAS pathway mutations. Alterations in canonical JMML driver genes, including PTPN11, NRAS and KRAS, typically had intermediate VAFs (typically in the 50–60% range) suggestive of major founding-clone events, while alterations in receptor tyrosine kinases and signaling regulators often had higher and more variable VAFs. The findings reveal the intricate clonal architecture of the dysregulation of the RTK-RAS pathway in JMML and show that both early driver and later subclonal events are important to the evolution of JMML.

### 3.7. Identification and Prioritization of Putative Driver Genes in RTK-RAS Pathway-Altered JMML

**Figure 7:**
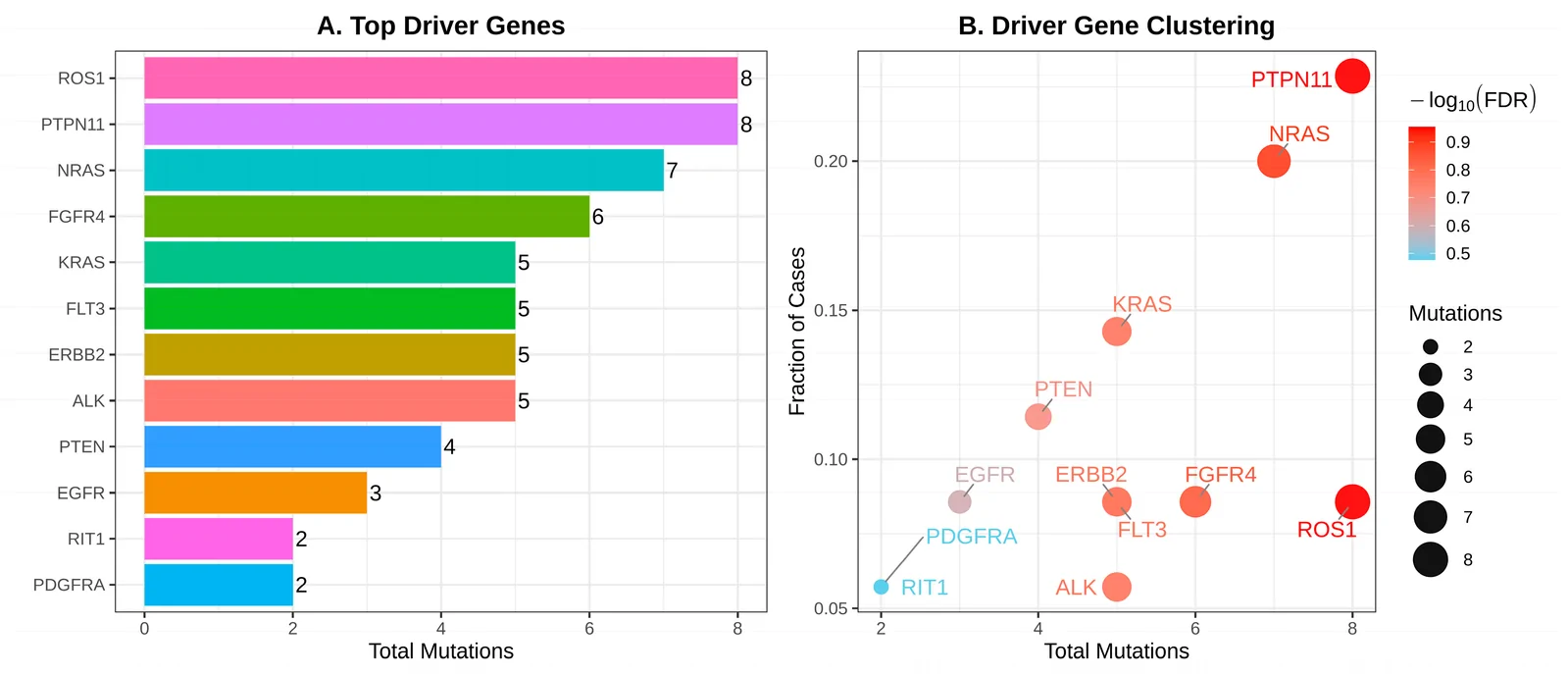
Driver gene landscape alterations in the RTK-RAS pathway in JMML. (**A**) Bar plot showing the most commonly mutated genes in the RTK–RAS pathway in the JMML cohort. (**B**) Driver gene clustering plot, showing the mutation frequency, the percentage of patients, and the *p*-value.

A driver gene analysis was performed to identify candidate driver genes involved in RTK-RAS pathway dysregulation in JMML (Figure 7). The analysis identified multiple genes that were repeatedly altered and showed evidence of positive selection, indicating that they play an important role in disease pathogenesis. The top-ranked driver genes are shown in Figure 7A. Of these, PTPN11 and ROS1 had the most driver mutation events (*n* = 8 each), followed by NRAS (*n* = 7) and FGFR4 (*n* = 6). Additional recurrent driver events were identified in KRAS, FLT3, ERBB2, and ALK (*n* = 5 each), while PTEN and EGFR showed four and three driver events, respectively. To further investigate the importance of these recurring events, driver gene clustering analysis was conducted (Figure 7B). Cluster plot analysis revealed that PTPN11 was the most significantly associated gene and also had the highest percentage of affected cases, indicating its central role in the biology of JMML. NRAS also showed a high mutation burden and a high frequency of mutations, consistent with its well-established role as a major driver gene in JMML. Other recurrently enriched genes, including ROS1, FGFR4, KRAS, FLT3, ERBB2, ALK, and PTEN, showed varying levels of recurrence and significance, indicating that both receptor tyrosine kinases and downstream signaling molecules contribute to disease development. Altogether, driver gene analysis identified a group of genes, including receptor tyrosine kinase genes and PTPN11 and NRAS and KRAS, which are frequently activated in the RTK-RAS pathway in JMML. The results of these studies further highlight the molecular diversity of JMML and underscore the pivotal role of dysregulated RTK-RAS signaling in disease pathogenesis.

### 3.8. Therapeutic Actionability and Drug–Gene Interaction Landscape of RTK-RAS Pathway Alterations in JMML

**Figure 8:**
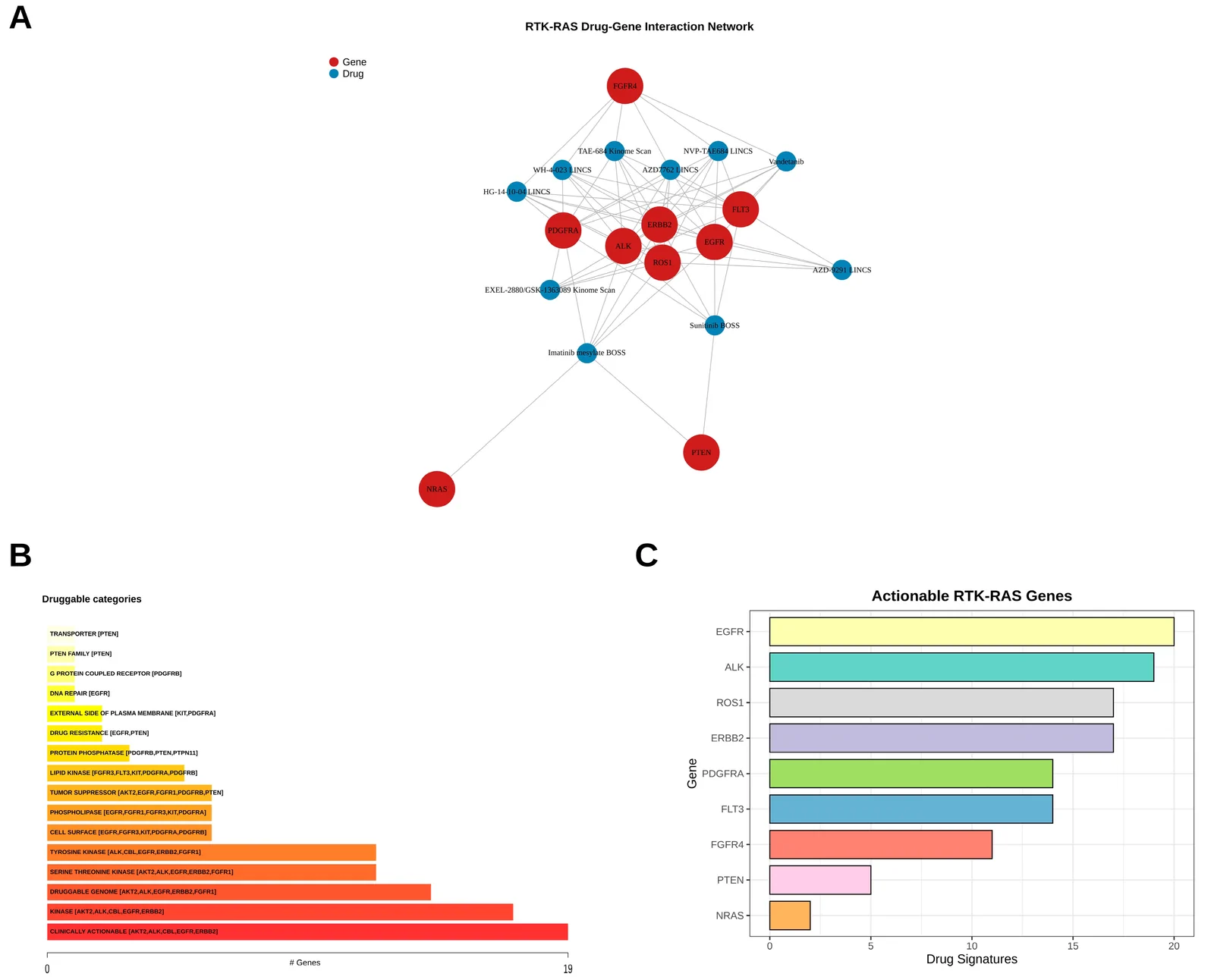
Druggability landscape of recurrent RTK-RAS pathway alterations in JMML. (**A**) Drug–gene interaction network showing the relationships between recurrently altered genes of the RTK-RAS pathway (red nodes) and therapeutic compounds (blue nodes). (**B**) Functional categories of druggable genes of the RTK-RAS pathway based on curated therapeutic categories. (**C**) Actionable RTK-RAS pathway genes quantified by the number of drug signatures associated with them.

To assess the translatability of recurrent RTK-RAS pathway alterations found in the JMML cohort, a comprehensive druggability analysis was carried out to determine genes that can be targeted by clinically available therapeutic agents (Figure 8). Recurrently altered genes in the RTK-RAS pathway showed high connectivity with various therapeutic agents in a drug–gene interaction network analysis (Figure 8A). The receptor tyrosine kinase genes, such as EGFR, ERBB2, ALK, ROS1, FGFR4, FLT3 and PDGFRA, showed the highest level of interaction with targeted compounds, which are known to be druggable oncogenic drivers. Multiple clinically relevant inhibitors, such as vandetanib, sunitinib, imatinib, and several investigational kinase inhibitors, were linked to these genes, suggesting therapeutic vulnerabilities in our cohort. Conversely, there were fewer direct drug interactions observed for canonical JMML driver genes, such as NRAS, reflecting previous difficulties with pharmacological targeting of RAS proteins. Actionable genes were functionally annotated and found to be enriched for a number of druggable molecular categories (Figure 8B). Clinically actionable genes were ALK, CBL, EGFR, ERBB2, followed by those in the kinase, druggable genome, and serine/threonine kinase groups. Further, the tyrosine kinase, cell surface protein, phospholipase, lipid kinase, protein phosphatase and tumor suppressor classes were enriched, suggesting that the alterations of the RTK-RAS pathway may impact a wide range of therapeutically relevant molecular functions. The number of drug signatures found to be associated with specific genes was also analyzed in order to quantify the therapeutic actionability. EGFR showed the highest number of drug signatures, followed closely by ALK, ROS1, and ERBB2 (Figure 8C). Other actionable targets comprised PDGFRA, FLT3 and FGFR4, with many therapeutic interactions. These results indicate that receptor tyrosine kinases are the most promising targets for therapeutic intervention in JMML. The identified drug–gene interactions included both clinically approved agents and experimental/research compounds. Several agents, including imatinib, sunitinib, vandetanib, and osimertinib (AZD9291), are clinically available for specific indications, whereas compounds such as TAE-684/NVP-TAE684 represent experimental inhibitors. Importantly, the identification of these compounds indicates potential therapeutic targetability of RTK-RAS-associated genes and does not imply their clinical approval or established efficacy for the treatment of JMML. Overall, the druggability analysis revealed that a substantial proportion of recurrent RTK-RAS pathway alterations identified in the JMML cohort are potentially actionable.

### 3.9. Mutational Signature Analysis of RTK-RAS Pathway Alterations in JMML

**Figure 9:**
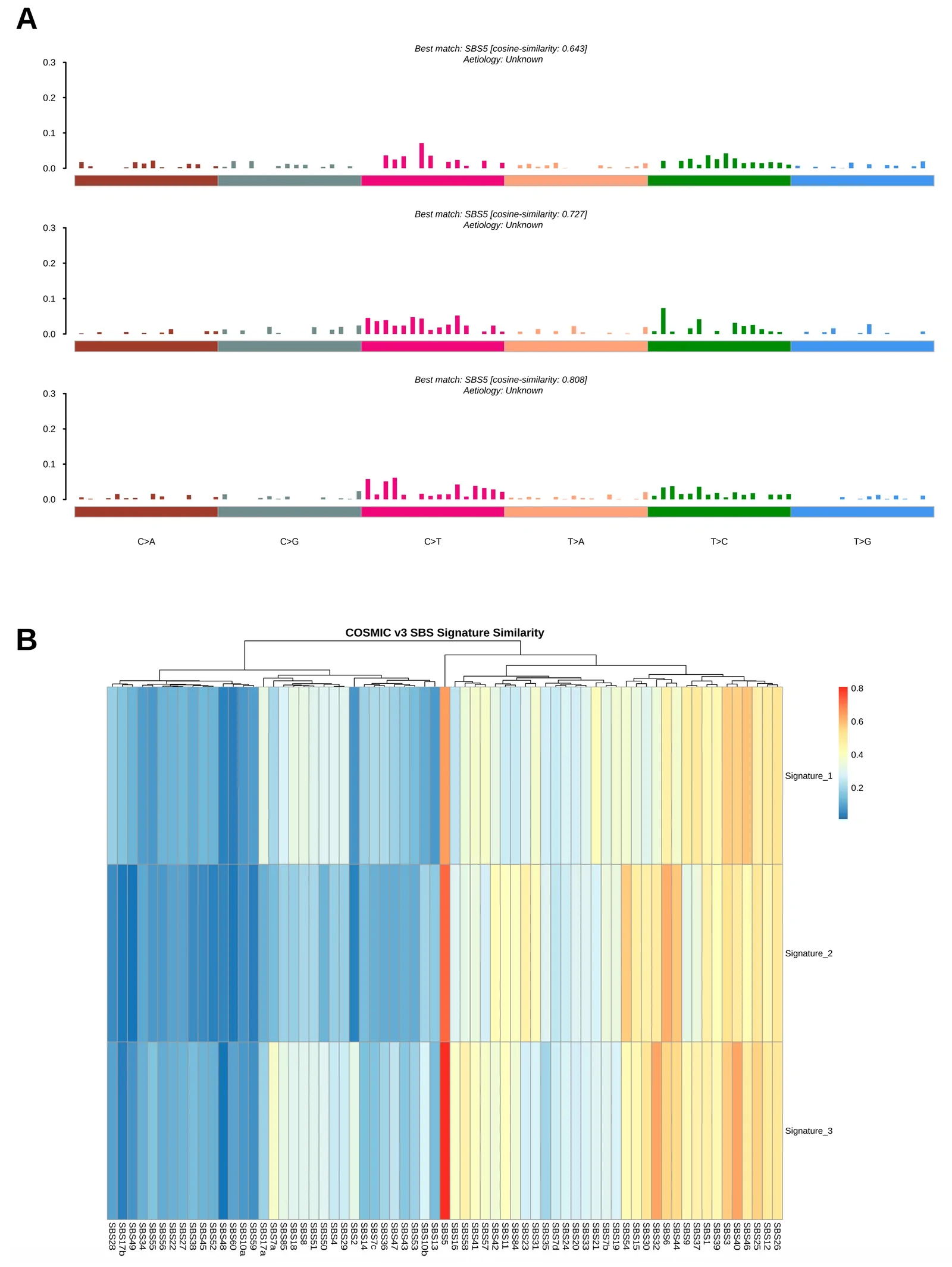
COSMIC mutational signature analysis of RTK-RAS pathway alterations in JMML. (**A**) De novo extracted mutational signatures from the RTK-RAS pathway mutations and their corresponding best matches to COSMIC v3 SBS signatures. (**B**) Heatmap showing cosine similarity between the extracted JMML mutational signatures and COSMIC v3 SBS reference signatures.

To investigate the mutational processes contributing to RTK-RAS pathway alterations in JMML, mutational signature analysis was performed using the COSMIC v3 single-base substitution (SBS) reference signatures (Figure 9). Mutational signatures were extracted from the cohort, and the three signatures were compared to known COSMIC SBS signatures using cosine similarity. The extracted signatures showed the highest similarity to COSMIC SBS5 (Figure 9A). The exact biological cause of SBS5 is still unknown, but it is thought to be a clock-like signature linked to endogenous mutational processes which build up over time. The extracted signatures revealed a strong bias towards C>T transitions with reduced levels of the other transitions, T>C, C>A, C>G, T>A, and T>G. All three signatures are very similar to SBS5 and indicate that the primary source of somatic mutations in RTK-RAS pathway genes in JMML is likely endogenous mutational processes, rather than exogenous mutagenic exposures. This was further supported by hierarchical clustering of cosine similarity scores among the COSMIC SBS signatures in the entire cohort (Figure 9B), which showed that the mutational patterns were primarily of the SBS5 type. The clustering analysis revealed that all three signatures were grouped as a single cluster, associated with the SBS5 pattern, suggesting that the most common RTK-RAS pathway mutations in JMML are caused by the same mutational process. Together, these results suggest that endogenous mutational processes associated with genomic aging and replication, such as SBS5-like signatures, are the most likely mutational processes driving RTK-RAS pathway mutations in JMML.

### 3.10. Functional Enrichment Analysis of RTK-RAS Pathway Genes in JMML

**Figure 10:**
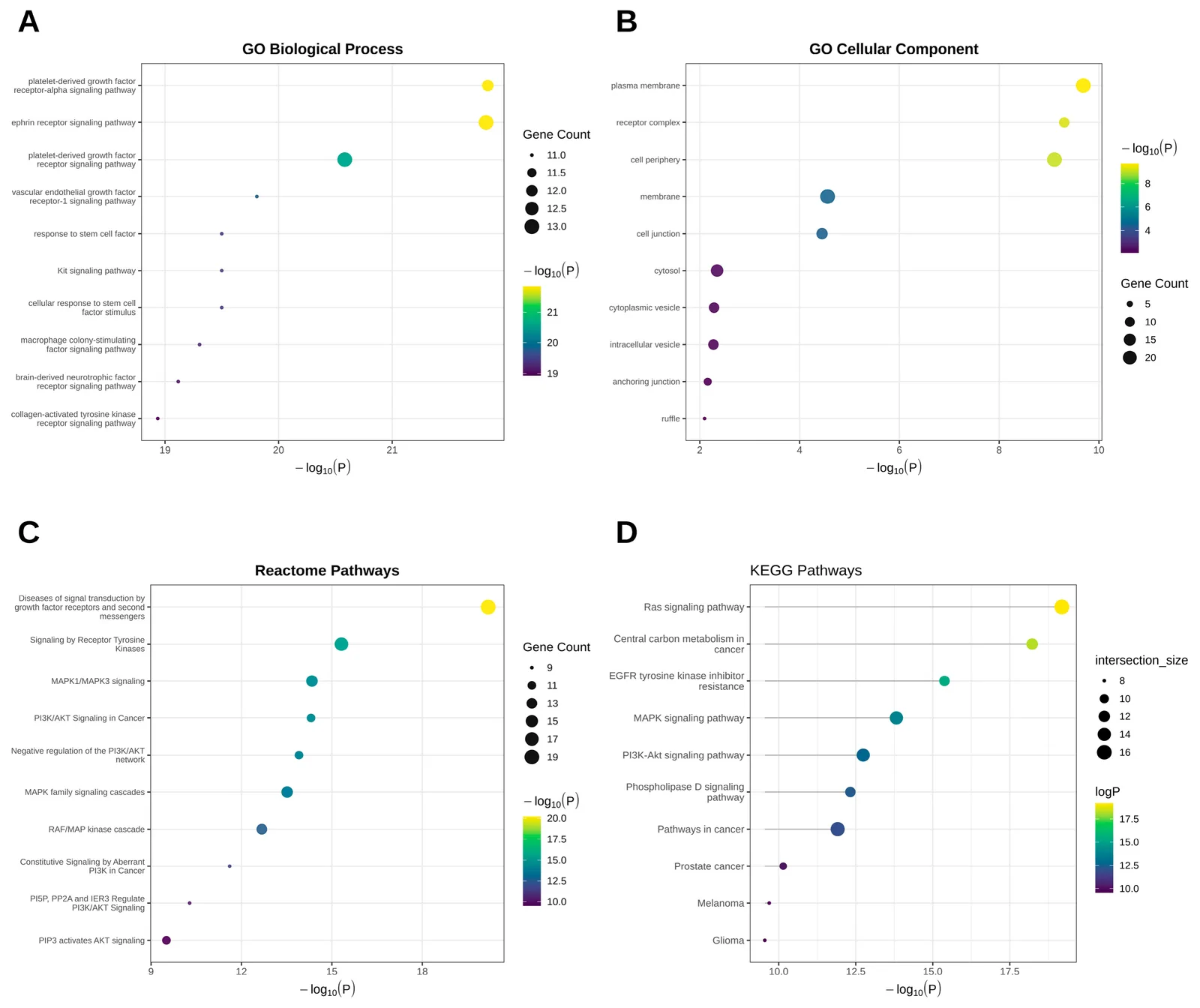
Functional enrichment analysis of recurrently altered RTK-RAS pathway genes in JMML. (**A**) Gene Ontology Biological Process (GO-BP) enrichment analysis. (**B**) Gene Ontology Cellular Component (GO-CC) enrichment analysis. (**C**) Reactome pathway enrichment analysis. (**D**) KEGG pathway enrichment analysis.

To provide biological insight into the functional significance of the altered RTK-RAS pathway genes, enrichment analysis was performed using Gene Ontology (GO), Reactome and KEGG pathway databases (Figure 10). Gene Ontology Biological Process (GO-BP) analysis revealed enrichment of pathways related to platelet-derived growth factor receptor alpha signaling, ephrin receptor signaling, and vascular endothelial growth factor receptor signaling, KIT signaling, response to stem cell factor, macrophage colony-stimulating factor receptor signaling, and collagen-activated tyrosine kinase receptor signaling, suggesting a widespread activation of growth-factor-dependent signaling pathways (Figure 10A). Gene Ontology Cellular Component (GO-CC) analysis showed that the changed genes were mostly related to plasma membrane, receptor complexes, cell periphery, and membrane-associated structures. Cell junctions, cytoplasmic vesicles and intracellular vesicles also showed significant enrichment, reflecting the localization of receptor tyrosine kinases and downstream signal transducers that play a role in cellular communication and signal transduction (Figure 10B). These were confirmed by Reactome pathway analysis, which showed that the most significantly enriched pathways were diseases of signal transduction by growth factor receptors and second messengers, signaling by receptor tyrosine kinases, MAPK1/MAPK3 signaling, PI3K/AKT signaling in cancer and MAPK family signaling cascades. Further, the canonical RTK-RAS downstream pathways are also enriched in JMML, such as the RAF/mutation-associated protein (MAP) kinase cascade and the phosphatidylinositol-3 kinase (PIP3)-activating protein (AKT) pathway, which further underscores their significance in the disease (Figure 10C). Consistent with these findings, the most significantly enriched pathways from KEGG pathway analysis were the Ras signaling pathway, MAPK signaling pathway, PI3K–Akt signaling pathway, EGFR tyrosine kinase inhibitor resistance, and phospholipase D signaling pathway. RTK-RAS signaling was also highly enriched in other cancer-related pathways such as prostate cancer, melanoma, glioma, and pathways in cancer (Figure 10D). The altered genes analyzed in this study were selected based on their involvement in the RTK-RAS signaling network; the enrichment analysis was not intended to independently establish RTK-RAS pathway involvement. Instead, it was performed to characterize the broader biological functions, interconnected signaling networks, and downstream pathways represented by the recurrently altered genes identified in our cohort. Together, these enrichment analyses suggest that genes that are recurrently altered in the JMML cohort are functionally clustered in closely connected receptor tyrosine kinase, RAS/MAPK, and PI3K/AKT signaling pathways. The increased activity of growth factor receptor signaling pathways and downstream oncogenic cascades indicate that RTK-RAS pathway dysregulation is a key molecular event in the pathogenesis of JMML.

## 4. Discussion

Juvenile myelomonocytic leukemia (JMML) is characterized by the constitutive activation of the RAS signaling pathway, and mutations in canonical genes such as PTPN11, NRAS, KRAS, NF1, and CBL are present in most patients [29,30]. However, there is growing evidence for further genetic changes that lead to disease heterogeneity, clonal evolution, and treatment responses [31,32]. In the present study, whole-exome sequencing of paired bone marrow and matched buccal swab samples from 35 JMML patients enabled a comprehensive characterization of alterations affecting the RTK-RAS signaling network. Our findings confirm the predominance of canonical RAS pathway lesions while also identifying recurrent alterations involving receptor tyrosine kinases and signaling regulators, highlighting the broader complexity of RTK-RAS pathway dysregulation in JMML. In line with previous genomic studies, PTPN11 was the most commonly altered gene in our cohort, followed by NRAS and KRAS [33,34]. Mutations involving residues within the N-SH2 domain of PTPN11, particularly E76K, D61Y, and D61V, have been shown to induce hypersensitivity of hematopoietic progenitors to granulocyte-macrophage colony-stimulating factor (GM-CSF) in JMML patients [35,36,37]. Experimental studies have shown that transduction of bone marrow or fetal-liver-derived mononuclear cells with the E76K mutant leads to a significant increase in erythroid burst-forming unit (BFU-E) colonies, a finding that has been associated with the increase in circulating erythroblasts in JMML patients [38]. Furthermore, macrophage progenitors carrying the E76K, D61Y or D61V mutation display hyperproliferative responses to GM-CSF and increased basal and sustained GM-CSF-induced ERK phosphorylation [35]. Mutations in NRAS and KRAS, especially G12 mutations, are known as early founder events in JMML and are associated with the aberrant proliferation of hematopoietic progenitors in the developmental stage corresponding to the origin of the disease [39,40]. Beyond canonical driver mutations, we identified recurrent alterations affecting receptor tyrosine kinases, such as PTEN, FLT3, ROS1, FGFR4 and ERBB2. The FLT3 T227M variant found in our cohort was previously shown to affect monomer-dimer interconversion of FLT3 and to modify the binding of sunitinib to the activated kinase domain [41]. In addition, we found the FGFR4 mutations V10L in the signal peptide region and P136L in the region between the two immunoglobulin-like domains, both of which could impact protein function [42,43]. Interestingly, the FGFR4-G388R variant was also observed in our cohort. This polymorphism has been shown in several studies to be associated with tumor progression and aggressiveness by increasing STAT3 signaling [44]. Together, these findings indicate that disordered receptor-mediated signaling is another mechanism that can lead to disease in JMML.

Somatic interaction analysis demonstrated that there was mutual exclusivity among genes of the canonical RAS pathway and positive relationships among receptor tyrosine kinase alterations. Mutations in PTPN11, NRAS, and KRAS have been reported as mutually exclusive in JMML, which further suggests that activation of one major RAS driver alone is enough to initiate the disease [22,45,46]. By contrast, co-occurring RTK mutations could be acquired during clonal evolution, offering further proliferative benefits and potentially contributing to treatment resistance [47,48,49]. Additionally, variant allele frequency analysis revealed that mutations in PTPN11, NRAS and KRAS typically had intermediate VAFs, suggesting founder-clone events. In contrast, RTK mutations were significantly more variable and, in some instances, of high allelic frequency, suggestive of the presence of additional mutations acquired as disease progresses and clonal heterogeneity. Previous studies have demonstrated that JMML progresses by the development of secondary lesions in epigenetic modifiers and signaling genes, which lead to subclonal expansion and to the risk of relapse after hematopoietic stem cell transplantation [50]. The heterogeneous VAF patterns observed in our cohort support this model of stepwise clonal evolution. Driver gene analysis further highlighted the central importance of PTPN11 and NRAS, along with receptor tyrosine kinase genes, as other possible driver genes. Interestingly, the two genes, ROS1 and FGFR4, showed recurrent clustering that suggested positive selection. These genes have not been studied extensively in JMML, but their well-established role in other malignancies makes it possible that they are involved as a cooperating oncogenic driver in JMML [51,52,53,54].

An important finding of our study is the identification of multiple therapeutically actionable alterations. A drug–gene interaction analysis revealed extensive interaction between recurrently altered receptor tyrosine kinases and clinically available inhibitors. Many interactions were observed between targeted compounds in genes like EGFR, ERBB2, ALK, ROS1, PDGFRA, and FLT3. However, these computationally derived drug–gene associations indicate potential druggability and should not be interpreted as evidence that the specific variants identified in our cohort are functionally targetable or predict drug sensitivity or clinical response in JMML. Interestingly, PTEN showed binding to sunitinib; previous research has demonstrated that sunitinib selectively killed SHP2-mutant leukemia cells, in a possible therapeutic approach for SHP2-mutant JMML [55]. Likewise, imatinib mesylate interacted with PDGFRA, ALK, ROS1, ERBB2 and EGFR. Imatinib has been shown to be effective in the treatment of myeloproliferative disorders with PDGFR rearrangements, such as JMML [56]. Allogeneic hematopoietic stem cell transplantation (HSCT) is still the sole curative treatment for JMML, but the risk of relapse is high and new therapeutic approaches are urgently needed [57]. However, promising results with MEK inhibitors like trametinib have been shown recently in patients affected by JMML with a RAS mutation [58,59]. In this context, the additional RTK alterations identified in our cohort represent potential candidates for future therapeutic investigation. The present computational drug–gene analysis is hypothesis-generating and does not establish therapeutic efficacy or clinical actionability of these alterations. Although combinations of MAPK pathway inhibitors and receptor tyrosine kinase-directed therapies may represent a potential area for future investigation in selected molecular contexts, the efficacy of such approaches requires validation in appropriate functional models and prospective clinical studies before consideration as a precision-medicine strategy. Mutational signature analysis showed a strong similarity to COSMIC SBS5, which is the most prevalent signature with an over-representation of C>T transitions. SBS5 is regarded as a clock-like signature linked to endogenous mutational processes and has been found in many different human tissues and malignancies [60]. The lack of signatures linked to exogenous mutagens supports the idea that the development of JMML is mainly influenced by intrinsic biological mechanisms, and not by environmental carcinogens. In previous genomic analyses of pediatric leukemia, the overall mutational burden is low and the number of mutational signatures is also limited [61]. Functional enrichment analysis showed strong enrichment of receptor tyrosine kinase signaling, MAPK cascade, PI3K-AKT pathway, and growth factor receptor-mediated signaling. These results are similar to those of previous transcriptomic and genomic analyses showing that hyperactivation of the RAS/MAPK and PI3K signaling pathways is a molecular hallmark of JMML [14,62,63]. A further indication of the extensive crosstalk between growth factor receptors and downstream signaling molecules is provided by the enrichment of platelet-derived growth factor receptor signaling and VEGF receptor signaling and stem-cell-factor-mediated pathways [64,65,66]. All of this suggests that JMML does not require single RAS mutations but instead involves a highly complex and interdependent network of signaling pathways. There are several limitations to the present study. First, the relatively small cohort size (*n* = 35), reflecting the rarity of JMML, may limit statistical power for detecting less common alterations and evaluating pairwise mutational associations. Several alterations occurred at low frequencies, resulting in sparse contingency tables and wide or infinite confidence intervals for some associations. Although four gene pairs remained statistically significant after correction for multiple comparisons, these findings should be considered exploratory and hypothesis-generating rather than evidence of biological cooperation or functional interaction. Second, buccal swab DNA was used as the matched non-tumor control. Although this supported discrimination between somatic and germline variants, constitutional mosaicism and contamination by hematopoietic cells cannot be completely excluded. Pathogenic or likely pathogenic germline predisposition variants were not systematically analyzed or reported separately, and future studies using alternative non-hematopoietic germline sources and dedicated germline analyses will be important. Third, VAF-based analyses were not adjusted for copy-number alterations, loss of heterozygosity, or tumor purity and were therefore interpreted only as descriptive measures of allelic representation rather than definitive estimates of cancer cell fraction or clonal architecture. Copy-number alterations, structural variants, and transcriptomic changes were also not investigated. Finally, the findings were derived primarily from genomic and computational analyses, without functional validation. Although recurrent RTK-RAS pathway-associated variants were prioritized using recurrence, database annotations, population frequency, and in silico predictions, these criteria do not establish functional driver activity. In particular, the biological significance of uncommon alterations in genes such as *ROS1*, *FGFR4*, *FLT3*, and *ERBB2* remains to be determined. These variants should therefore be considered candidate or recurrent alterations rather than definitively established JMML drivers. Validation in larger independent cohorts and functional studies will be required to determine their biological and clinical significance. Despite these limitations, our study provides a comprehensive characterization of high-confidence alterations affecting the RTK-RAS signaling network in this JMML cohort. The findings further support the importance of dysregulated RTK-RAS signaling in JMML and identify several less frequently reported RTK-RAS pathway-associated alterations that warrant further investigation. However, the biological and clinical significance of these uncommon variants requires validation in larger independent cohorts and functional studies. These findings provide a basis for future investigations aimed at clarifying the functional relevance of these alterations and their potential implications for JMML biology and therapeutic development.

## 5. Conclusions

Whole-exome sequencing revealed a complex landscape of RTK-RAS pathway alterations, highlighting the central role of aberrant receptor tyrosine kinase and RAS signaling in JMML pathogenesis. While canonical driver genes such as PTPN11, NRAS, and KRAS remained the predominant alterations, recurrent mutations involving ROS1, FGFR4, FLT3, ERBB2, EGFR, ALK, and PTEN expanded the spectrum of signaling abnormalities associated with JMML. Protein domain mapping and driver gene analyses identified functionally relevant regions of mutation clustering, while somatic interaction and variant allele frequency analyses showed evidence of clonal heterogeneity and cooperative evolution of receptor tyrosine kinase alterations. The mutational signature analysis showed that mutational processes of the SBS5-like group were predominant, and functional enrichment analyses showed that interconnected RTK, RAS/MAPK, and PI3K-AKT signaling pathways were involved. Importantly, analysis of drug–gene interactions identified potentially actionable targets, indicating potential therapeutic interventions beyond conventional therapy. Taken together, these results offer a thorough genomic understanding of the dysregulated RTK-RAS pathway in JMML and serve as a foundation for precision medicine approaches and future functional experiments to enhance the clinical outcomes in children with this aggressive disease.

## Data Availability

The data supporting the findings of this study are not publicly available due to ethical and privacy restrictions but are available from the corresponding author upon reasonable request.

## Supplementary Materials

The following supporting information can be downloaded at: https://www.mdpi.com/article/10.3390/medsci14050583/s1, Table S1: Detailed annotation of RTK-RAS pathway-associated variants identified by whole-exome sequencing in 35 JMML patients. Table S2: Detailed pairwise somatic interaction analysis of RTK-RAS pathway-associated genes in 35 patients with JMML. Table S3: Clinical, hematological characteristics of 35 patients with juvenile myelomonocytic leukemia (JMML).

## Abbreviations

The following abbreviations are used in this manuscript:

JMML	Juvenile myelomonocytic leukemia
RTK	Receptor tyrosine kinases
HSPCs	Haematopoietic stem/progenitor cells
MDS	Myelodysplastic syndromes
WHO	World Health Organization
ICC	International Consensus Classification
HSCT	Hematopoietic stem cell transplantation
WES	Whole-exome sequencing
BM	Bone marrow
SNVs	Single nucleotide variants
GATK	Genome Analysis Toolkit
BWA	Burrows–Wheeler Aligner
SNV	Single nucleotide variants
MAF	Mutation Annotation Format
KEGG	Kyoto Encyclopedia of Genes and Genomes
VAF	Variant allele frequencies
FDR	False discovery rate
NMF	Negative matrix factorization
SBS	Single-base substitution
